# Utilization and outcomes of COVID-19 positive donors in pediatric kidney transplantation—a population-based study

**DOI:** 10.1007/s00467-026-07362-y

**Published:** 2026-06-06

**Authors:** Saritha Ranabothu, Michael D. Evans, Sarah J. Kizilbash

**Affiliations:** 1 Department of Pediatrics, Pediatric Nephrology, Arkansas Children’s Hospital, University of Arkansas Medical Sciences, Little Rock, AR USA; 2 Clinical and Translational Science Institute, University of Minnesota, Minneapolis, MN USA; 3 Department of Pediatrics, University of Minnesota, Minneapolis, MN USA

**Keywords:** Donor COVID, Pediatric kidney transplant, Outcomes, Acceptance

## Abstract

**Background:**

COVID-19 infection has been associated with significant morbidity and mortality across all age groups, yet data on pediatric kidney transplant outcomes associated with COVID-19 positive (COVID +) donors remain limited.

**Methods:**

Using the Scientific Registry of Transplant Recipients, we identified 143 pediatric kidney recipients (< 18 years) of COVID + donors transplanted between September 2020 and February 2025 and compared them with 1808 recipients of COVID-19 negative (COVID −) donors using propensity score weighting to account for transplant year, age at transplant, sex, race, human leukocyte antigen mismatch, prior transplant, and immunosuppression.

**Results:**

Among 1940 pediatric recipients, 7.3% received kidneys from COVID + donors, with utilization increasing from 2.5% in 2020 to 10.5% in 2025. No statistically significant differences were observed in patient survival (HR 0.83, 95% CI 0.10–6.73, *p* = 0.86) and overall graft failure (HR 1.35, 95% CI 0.67–2.73, *p* = 0.41) between COVID + and COVID − groups over a median follow-up of 1.2 years. Delayed graft function (7.7% vs. 7.2%) and median initial hospital stay (8.0 vs. 8.0 days) were also comparable.

**Conclusions:**

The use of COVID + donors for pediatric kidney transplantation has increased over time. The posttransplant outcomes are similar between COVID + and COVID − pediatric recipients, supporting the use of COVID + donors in this population.

**Graphical Abstract:**

A higher resolution version of the Graphical abstract is available as Supplementary information

**Figure:**
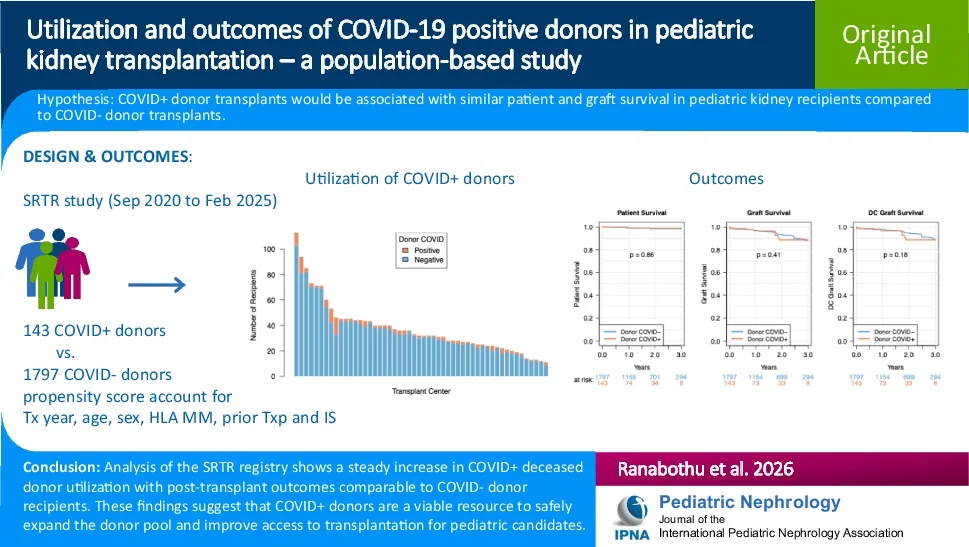

**Supplementary Information:**

The online version contains supplementary material available at https://doi.org/10.1007/s00467-026-07362-y.

## Introduction

Kidney transplantation is associated with better survival, improved growth, and better neurocognitive development compared to chronic dialysis in children with kidney failure [1]. However, kidney transplantation in children is often delayed due to widespread organ shortages. The number of pediatric candidates on the deceased donor waitlist in the United States increased from 2199 in 2012 to 2956 in 2023, demonstrating the impact of organ shortages on children. Of the 1079 candidates removed from the waitlist in 2023, only 550 (69.5%) received a deceased donor transplant, 241 (30.5%) received a living donor transplant, 14 (1.3%) died while waiting, and 10 (0.3%) became too sick for transplant. These figures highlight a 16.1% decline in the deceased donor kidney transplantation rate in children over the past decade [2], emphasizing the urgent need to increase organ availability for children.

The COVID-19 pandemic, caused by the SARS-CoV-2 virus (severe acute respiratory syndrome coronavirus 2), profoundly impacted global healthcare, including solid organ transplantation. Early in the pandemic, organ transplantation volumes declined due to concerns about donor-derived transmission, recipient vulnerability, and strains on the health system. As evidence accumulated, alongside widespread vaccination and the emergence of less severe variants, the perceptions of risk shifted. Guidance from the Organ Procurement and Transplantation Network (OPTN) shifted to cautiously support consideration of organs from COVID + donors following individualized risk assessment and appropriate testing, particularly for non-lung organs [3, 4]. Coinciding with the policy shift, the utilization of abdominal organs from COVID + donors in adults increased, and studies showed favorable outcomes (no difference in short-term graft or patient survival) and no significant donor-derived COVID-19 transmission for non-lung recipients [5, 7]. In contrast to adult data, the utilization of COVID + donors for pediatric kidney transplant recipients and outcomes associated with their use remain unexamined; pediatric data are sparse and limited to case reports with short follow-up periods [8, 9]. A 2022 survey of 154 pediatric solid organ transplant centers in the United States found that only 31 centers (20%) accepted organs from COVID + donors, highlighting the hesitancy among pediatric providers and underscoring the need for more outcome data [10].

Using the Scientific Registry of Transplant Recipients (SRTR), we evaluated COVID + donor acceptance rates for pediatric kidney transplantation in the United States. We also compared kidney transplant outcomes between COVID + and COVID − pediatric kidney transplant recipients. To account for the pediatric priority on the waiting list and shorter wait times, we compared the effect of a COVID + donor transplant on patient survival versus remaining on the waiting list for a COVID − donor offer. Considering the favorable effect of COVID + donor transplants on adult outcomes, we hypothesized that COVID + donor transplants would be associated with similar patient and graft survival in pediatric kidney recipients compared to COVID − donor transplants. Furthermore, considering the survival benefit of timely transplants in children compared to remaining on the waiting list [11, 12], we hypothesized a survival benefit of COVID + transplants over the waitlist alternative. To our knowledge, this is the first population-based study to evaluate the effects of COVID + donor transplants on survival in children with kidney failure.

### Methods

This research was determined to be “non-human subject” research by the University of Arkansas for Medical Sciences (UAMS) Institutional Review Board (Reference #: 300039). A copy of the formal documentation is available as Supplementary Material.

#### Data source

This study used data from the SRTR. The SRTR data system includes data on all donors, wait-listed candidates, and transplant recipients in the US, submitted by the members of the OPTN. The Health Resources and Services Administration (HRSA), U.S. Department of Health and Human Services, provides oversight to the activities of the OPTN and SRTR contractors [13].

#### Study population

Pediatric kidney transplant recipients, defined as individuals aged 18 years or younger at the time of transplant, who underwent transplantation in the United States between September 2020 and February 2025. Inclusion was restricted to recipients of deceased donor organs where the donor underwent COVID-19 testing.

**Fig. 1:**
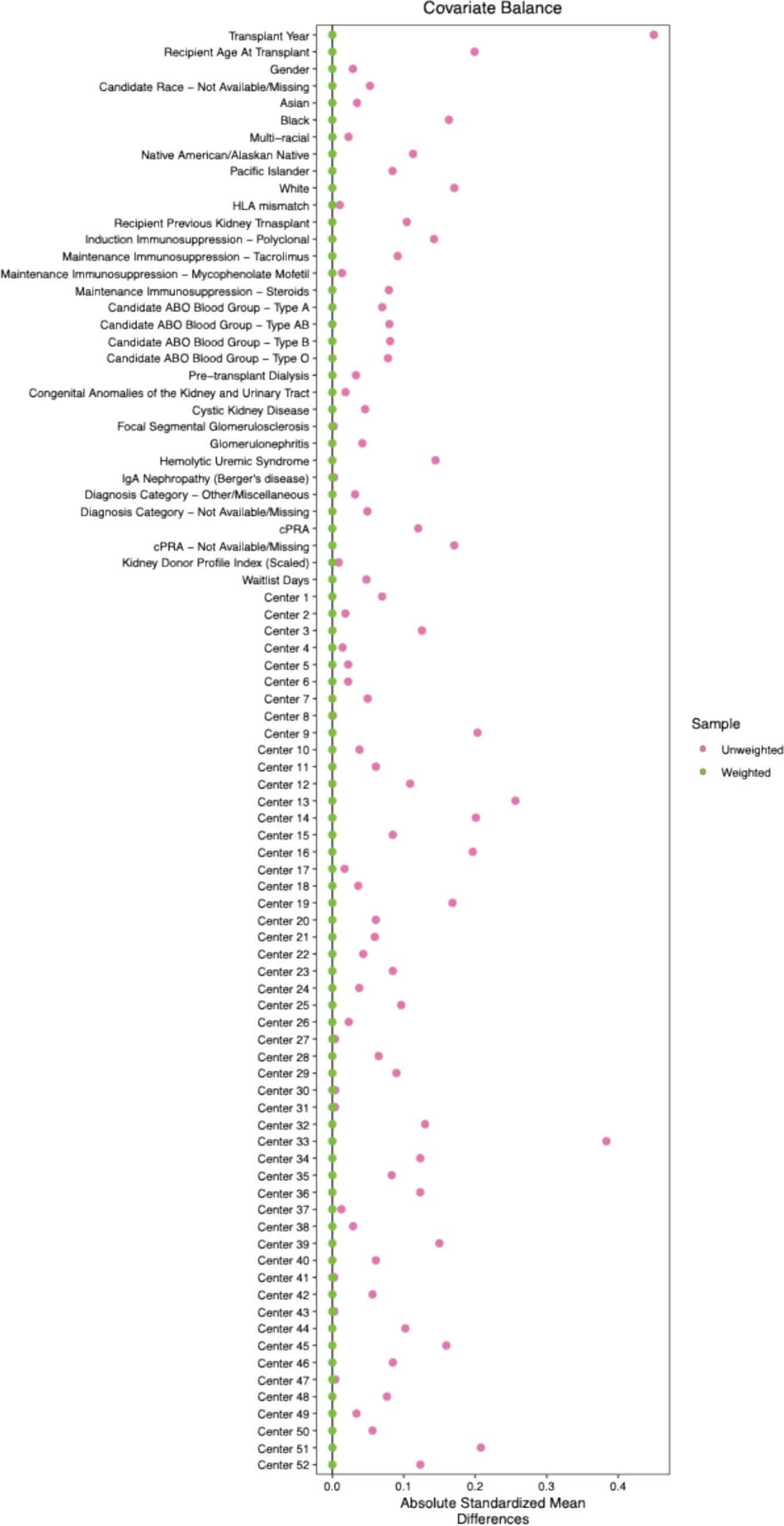
Covariate balance between COVID + and COVID − recipients before and after weighting

To compare transplant outcomes between COVID + and COVID − recipients, we divided the recipients into two groups: recipients of COVID + donors (*n* = 143) and recipients of COVID − donors (*n* = 1797). We used propensity score weighting to create a weighted comparison group of COVID − recipients with characteristics similar to those of COVID + recipients[14]. Covariate-balancing propensity score weights [15] were computed using a logistic regression model of COVID status (positive vs. negative) regressed on variables, including year of transplant, age at transplant, recipient sex, recipient race, number of HLA mismatches, prior kidney transplant, recipient’s blood type, pretransplant dialysis, cause of kidney failure, waitlist time, calculated Panel Reactive Antibody (cPRA), induction immunosuppression, maintenance immunosuppression, and transplant center. Covariate balance before and after weighting was assessed using the absolute standardized mean difference (SMD) between COVID + and COVID − recipients (Fig. 1). Our final analysis cohort of 1940 recipients included all pediatric deceased donor kidney transplants from centers with at least one COVID + donor and at least one COVID − donor among qualifying recipients. Eleven patients were excluded due to unknown COVID status. Of note, no COVID + living donor transplants were reported in SRTR.

#### Study variables

Our variables of interest were as follows: age at transplant, recipient sex and race, pretransplant dialysis/preemptive transplant, cause of kidney failure, prior transplants, number of human leukocyte antigen (HLA) mismatches, cPRA, blood type, transplant year, Kidney Donor Profile Index (KDPI), induction and maintenance immunosuppression, and, where applicable, causes of graft loss and causes of death. Our primary exposure of interest was the donor’s COVID status.

SARS-CoV-2 testing was primarily conducted using nucleic acid detection methods (real-time-polymerase chain reaction), which were utilized for 99.7% (*n* = 1934) of the cohort. Other testing methods included antigen testing (3.1%), antibody testing (2.7%), and other unspecified methods (2.4%).

Regarding specimen collection, upper respiratory samples (e.g., nasopharyngeal swabs) and lower respiratory samples (e.g., bronchoalveolar lavage or tracheal aspirate) were the most frequent, representing 87.3% and 84.5% of the donors, respectively. A small percentage of results were derived from blood samples (2.8%) or other sources (1.4%). Because individual donors could have multiple test results reported, the cumulative percentages exceed 100%. Within the SRTR dataset, a “COVID-positive” designation is derived from standardized clinical testing performed during the formal donor evaluation and procurement process, typically within 72 h prior to organ recovery. Consequently, these data represent active or acute infections at the time of donor death rather than a remote historical record of prior infection.

#### Study outcomes

Our outcomes of interest were patient survival, overall graft survival, and death-censored graft survival. We also examined delayed graft function (DGF) (defined as dialysis within the first week post-transplant) and the length of initial hospital stay (defined as the interval between the date of transplant and the date of discharge for the transplant surgery-related hospitalization). For patient survival, we followed recipients from the date of transplant to the earliest of the dates of death or the end of SRTR follow-up. For graft survival, we followed recipients from the date of transplant to the earliest of the dates of graft loss, death, or the end of SRTR follow-up.

Donor-derived transmission was not examined, as these data were unavailable.

#### Statistical analysis

We compared categorical and continuous variables between COVID + and COVID − recipients using chi-squared and Wilcoxon tests, respectively. We used the Kaplan–Meier method to estimate patient survival, graft survival, and death-censored graft survival, and Cox proportional hazards models to estimate hazard ratios with 95% confidence intervals. The proportional hazards assumption was assessed by visual inspection of the Kaplan–Meier curves and complementary log–log plots. The Z tests for proportionality of hazards did not provide significant evidence of non-proportionality between COVID − and COVID + for patient (*p* = 0.75), graft (*p* = 0.18), or death-censored graft (*p* = 0.19) survival. Delayed graft function was compared by the COVID status using the weighted chi-squared test. The length of hospital stay was compared by the COVID status using the weighted Wilcoxon rank sum test. The covariate-balancing propensity score was used to calculate inverse probability of treatment weights. Weighted models were fit using robust standard errors. Missingness rates were 0.0% for all characteristics except primary diagnosis (0.2%) and cPRA (17.7%). Missing data were addressed in the propensity score model using the indicator variable method. First, for each variable with missingness, a new missingness indicator variable is created which takes the value 1 if the original covariate is NA and 0 otherwise. The missingness indicators are added to the model formula as main effects. The missing values in the covariates are then replaced with the covariate medians (this value is arbitrary and does not affect estimation). The weight estimation then proceeds with this new formula and set of covariates. The covariates output in the resulting weightit object will be the original covariates with the NAs.

Causes of death and causes of graft failure were compared between COVID + and COVID − using Fisher’s exact test.

To evaluate the survival benefit of a COVID + donor transplant versus remaining on the waiting list for a COVID − donor, we used sequential stratification [16]. For each COVID + donor transplant case, we created a control group consisting of all pediatric candidates on the waitlist on their respective case’s date of transplant. We followed each case and its corresponding control group from the case’s transplant date to the earliest of the dates of death or the last follow-up. We censored the follow-up on the waitlist control’s date of transplant if the control subsequently received a COVID + donor transplant or a living-donor transplant. Similarly, the follow-up was censored if the waitlist control was removed from the waitlist without a transplant. Each case with its corresponding control group constituted a separate “experiment” (also known as “stratum” or “landmark”). Waitlist control patients could be part of multiple strata. Waitlist candidates could serve as both cases (in one stratum) and controls (in one or more other strata) if they received a COVID + deceased-donor kidney. We compared patient survival between cases and control groups using stratified Cox models, with cluster-robust variance estimates to account for patients appearing in more than one stratum. Models were adjusted for age at the case’s transplant date, gender, race, blood type, and pretransplant dialysis.

Analyses were performed using R[17] version 4.4.2, including the packages WeightIt [18] version 1.3.2 for covariate-balancing propensity score weights, survey [19] version 4.4–2 for weighted versions of Kaplan–Meier, Cox models, and chi-squared tests, and Wilcoxon tests, cobalt [20] version 4.5.5 for balance assessment, and transplantr [21] version 0.2.0 for calculating KDPI. A two-sided *p*-value of < 0.05 was considered statistically significant.

## Results

### Donor utilization and study population

During the study period, a total of 68,717 deceased donors (134,929 kidneys) were considered nationally. Among these, 4948 donors (9609 kidneys) were identified as COVID-19 positive (COVID +). Of the COVID + donor pool, 3591 donors (72.6%) were accepted for transplantation, resulting in 6530 transplanted kidneys, while 1606 donors (2542 kidneys; 26.5%) were discarded.

From this cohort, our final study population consisted of 1940 pediatric kidney transplant recipients, of whom 143 (7.4%) received organs from COVID + donors and 1797 (92.6%) from COVID − donors. The median follow-up time for the entire cohort was 1.2 years (IQR, 0.6–2.8). Follow-up duration was comparable between groups, with a median of 1.2 years (IQR, 0.6–2.9) for COVID− recipients and 1.0 years (IQR, 0.5–2.0) for COVID + recipients.

### Recipient and donor characteristics

Table 1 presents the demographic and clinical characteristics of the study cohort. We observed no significant differences in sex, race, pretransplant dialysis, underlying cause of kidney failure, HLA mismatches, immunosuppression, ABO blood type, or the median KDPI between COVID + and COVID − recipients. The COVID + recipients were older compared to COVID − recipients (median age in years, 14.0 vs. 13.0; *p* = 0.04). COVID + recipients demonstrated a higher rate of prior transplants (12.0% vs. 8.0%), a higher cPRA (median, 0.02 vs. 0.0), and a shorter waitlist time (median in days, 183 vs. 206), however, these differences did not reach statistical significance.

**Table 1:** Demographic and clinical characteristics of kidney recipients from COVID + and COVID − donors

		Unadjusted			Adjusted		
			COVID + vs. COVID −			COVID + vs. COVID − (weighted)	
	COVID +	COVID −	SMD	*p*-value	COVID − (weighted)	SMD	Missing %
Number of recipients	143	1797			1797		
Effective sample size	143	1797			771		
Year, median [IQR]	2023 [2022, 2024]	2022 [2021, 2024]	0.40	< 0.001	2023 [2022, 2024]	0.00	0.0
Age at transplant, median [IQR]	14 [10, 16]	13 [8, 16]	0.19	0.05	14 [10, 16]	0.00	0.0
Male gender (%)	57%	59%	0.03	0.81	57%	0.00	0.0
Race (%)			0.27	0.16		0.00	0.0
Unknown	1%	1%			1%		
Asian	6%	6%			6%		
Black	27%	19%			27%		
Multi	3%	3%			3%		
Native	0%	1%			0%		
Pacific	2%	1%			2%		
White	61%	69%			61%		
Number of HLA mismatches, median [IQR]	5 [4, 5]	5 [4, 5]	0.01	0.69	5 [4, 5]	0.00	0.0
Previous KI Tx (%)	12%	8%	0.11	0.22	12%	0.00	0.0
Induct_poly (%)	73%	66%	0.14	0.14	73%	0.00	0.0
Maint_tacrolimus(%)	97%	95%	0.08	0.49	97%	0.00	0.0
Maint_MMF (%)	94%	93%	0.01	1.00	94%	0.00	0.0
Maint_steroids (%)	53%	57%	0.08	0.41	53%	0.00	0.0
Recipient blood type (%)			0.14	0.46		0.00	0.0
A	27%	30%			27%		
AB	5%	3%			5%		
B	10%	13%			10%		
O	58%	54%			58%		
Pretransplant dialysis (%)	74%	73%	0.03	0.78	74%	0.00	0.0
Primary diagnosis (%)			0.21	0.76		0.00	0.2
CAKUT	30%	29%			30%		
Cystic kidney	8%	6%			8%		
FSGS	10%	10%			10%		
GN	10%	9%			10%		
HUS	0%	2%			0%		
IgA nephropathy	2%	2%			2%		
Other	39%	41%			39%		
cPRA, median [IQR]	0.02 [0.00, 0.32]	0.00 [0.00, 0.25]	0.12	0.05	0.00 [0.00, 0.35]	0.00	17.7
KDPI, median [IQR]	3 [1, 10]	4 [1, 10]	0.01	0.54	3 [1, 10]	0.00	0.0
Wait time in days, median [IQR]	183 [62, 426]	206 [71, 447]	0.05	0.48	187 [65, 410]	0.00	0.0
(Transplant center)			0.74	0.002		0.00	0.0

*SMD *(absolute) standardized mean difference, *COVID +* coronavirus disease positive, *COVID −* coronavirus disease negative, *IQR* interquartile range, *HLA* human leucocyte antigen, *KI Tx* kidney transplant, *Poly* polyclonal immunosuppression, *MMF* mycophenolate mofetil, *CAKUT* congenital anomalies of kidney and urinary tract, *FSGS* focal segmental sclerosis, *GN* glomerulonephritis, *HUS* hemolytic uremic syndrome, *cPRA* calculated panel reactive antibody, *KDPI* kidney donor profile index

### Transplant outcomes

#### Delayed graft function

After weighting for year of transplant, age at transplant, recipient sex, recipient race, number of HLA mismatches, prior kidney transplant, recipient’s blood type, pretransplant dialysis, cause of kidney failure, waitlist time, cPRA, induction immunosuppression, maintenance immunosuppression, and transplant center, we observed no difference in the incidence of DGF between COVID + and COVID − groups (7.2% vs. 7.7%; *p* = 0.82).

#### Length of initial hospital stay

The length of hospital stay was similar between COVID + and propensity score-weighted COVID − recipients (median days, 8 [IQR, 6, 11] vs. 8 [IQR 6, 12]; *p* = 0.52).

#### Graft survival

**Fig. 2:**
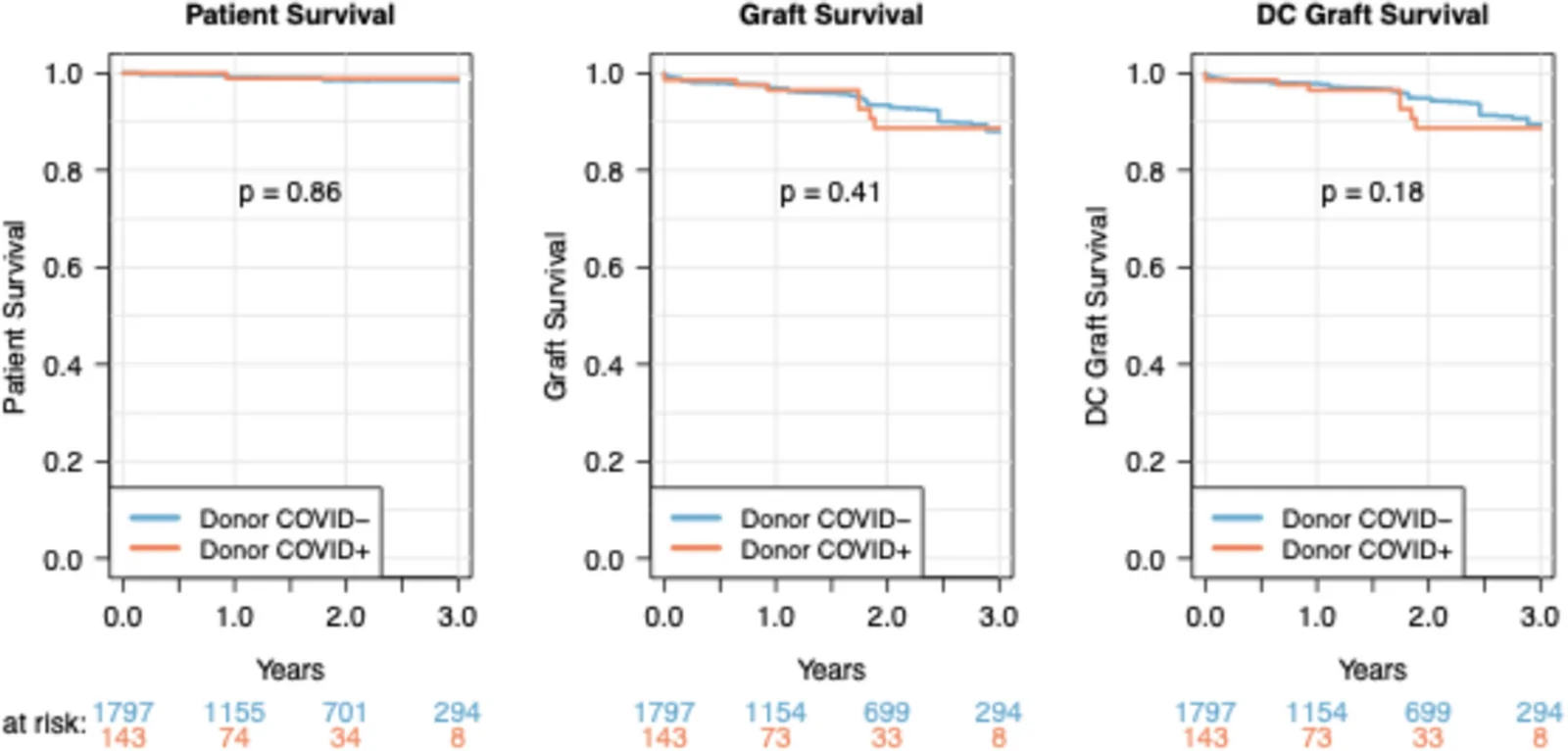
Patient, graft and death-censored graft survival of COVID + and propensity score-weighted COVID − pediatric kidney transplant recipients. DC: death censored; COVID +: coronavirus disease positive; COVID −: coronavirus disease negative

We found no difference in overall graft survival (HR, 1.35; 95% CI, 0.67–2.73; *p* = 0.41) and death-censored graft survival (HR, 1.63; 95% CI, 0.80–3.32; *p* = 0.18) between COVID + and propensity score weighted COVID − recipients (Fig. 2).

The 3-year graft survival rates for COVID + and propensity score-weighted COVID − recipients were 88.7% and 88%, respectively. Similarly, the 3-year death-censored graft survival for COVID + and propensity score-weighted COVID − recipients was 88.7% and 89.4%, respectively.

The causes of graft losses between the COVID + and COVID − recipients were similar (*p* = 0.88) (Table 2).

**Table 2:** Causes of graft loss and death in COVID + recipients vs. COVID − recipients

Category	COVID +	COVID −	*p*-value
**Number of deaths**	1/143 (0.69%)	13/1797(0.72%)	
**Causes of death**			0.29
Unknown	0	1	
Other	0	3	
Cardiovascular	0	1	
Cerebrovascular	0	1	
Trauma/motor vehicle accident	0	3	
Other trauma	0	2	
Respiratory failure	1	0	
Multiple system organ failure	0	2	
**Number of graft failures**	9/143 (6.2%)	70/1797 (3.9%)	
**Causes of graft failure**			0.88
Hyperacute rejection	0	1	
Graft thrombosis	2	9	
Surgical complications	0	2	
Primary non-function (graft never functioned post-transplant)	0	2	
Other	0	4	
Unknown	7	52	

*COVID +* coronavirus disease positive, *COVID −* coronavirus disease negative

#### Patient survival

We observed no statistically significant difference in patient survival between COVID + and propensity score-weighted COVID − recipients (HR, 0.83; 95% CI, 0.10–6.73; *p* = 0.86) (Fig. 2).

The 3-year patient survival was 98.9% for COVD + and 98.4% for COVID − recipients.

There was no statistically significant difference in the underlying causes of death between the groups (*p* = 0.29). One death among COVID + recipients was ascribed to respiratory failure, while two deaths in COVID − recipients were attributed to multiorgan failure (Table 2).

#### S﻿urvival associated with COVID + transplants versus remaining on the waiting list

When we examined the survival associated with COVID + transplant compared to remaining on the waiting list for a COVID – offer, we observed a trend towards improved survival (HR, 0.33; 95% CI, 0.05, 2.38; *p* = 0.27); however, the difference did not reach statistical significance.

The difference remained insignificant after adjusting for age at transplant, race, sex, pretransplant dialysis, recipient’s blood type, and transplant center (adjusted HR, 0.25; 95% CI, 0.03, 1.84; *p* = 0.17).

#### Patterns of COVID + donor utilization

**Fig. 3:**
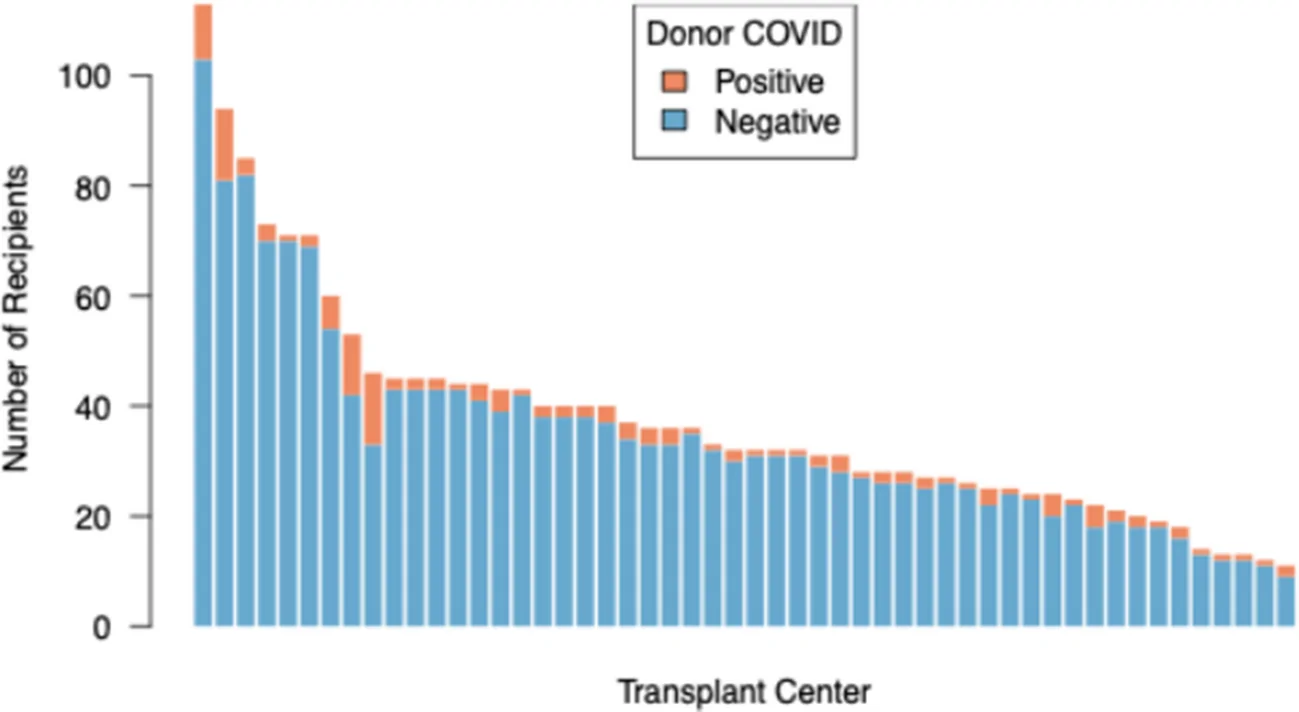
COVID + deceased donor kidney transplantation across pediatric kidney transplant centers. COVID +: coronavirus disease positive; COVID −: coronavirus disease negative

The percentage of pediatric kidney transplants utilizing COVID + deceased donors in the United States rose substantially during the study period, increasing from 2.0–2.6% annually in 2020 and 2021 to 9.1–10.5% from 2022 onward (Table 3). Furthermore, centers of all sizes (low- and high-volume centers) performed COVID + decreased-donor pediatric kidney transplantation (Fig. 3).

**Table 3:** Acceptance of COVID + donors over the years

Year of transplant	Recipients	Donor COVID +	
	*N*	*N*	%
All	1940	143	7.4%
2020	115	3	2.6%
2021	461	9	2.0%
2022	392	41	10.5%
2023	429	40	9.3%
2024	477	44	9.2%
2025	66	6	9.1%

Total *N* = 1940 includes all recipients with age at transplant < = 18y, transplant date between 2020–09-13 and 2025–02-25, kidney alone, deceased donor (there were 0 COVID + living donors), from all transplant centers with at least one COVID + donor and at least one COVID − donor among qualifying recipients. *COVID +* coronavirus disease positive, *COVID −* coronavirus disease negative

## Discussion

The utilization of COVID + donors for pediatric deceased-donor kidney transplantation in the United States increased substantially over the study period, exceeding 9% of all pediatric deceased-donor transplants performed between 2022 and 2025. We observed no statistically significant difference in patient survival, graft survival, DGF rates, and the length of initial hospital stay between COVID + and propensity score-weighted COVID − groups. Our study represents the first population-based study to demonstrate comparable outcomes between COVID + and COVID − pediatric deceased-donor kidney transplant recipients, providing evidence to inform acceptance decisions for children.

The use of COVID + deceased donors for pediatric kidney transplantation has increased over time. This mirrors the trend seen in adults, where the initial reluctance to use these donors has diminished over time [7]. Interestingly, we found no difference in COVID + acceptance practices across transplant centers by transplant volume, suggesting that acceptance decisions may be influenced by center-specific policies, local practices, or individual physician judgment rather than by center volume or transplant experience.

Existing pediatric data on outcomes associated with COVID + donor transplantation are sparse. A report of 2 pediatric kidney recipients of COVID + donors described no donor-derived COVID-19 transmission and functioning grafts at 90 days posttransplant [8]. Another series of 2 pediatric COVID + donors with 7 adult and pediatric solid organ recipients (excluding lungs) reported no evidence of COVID transmission and 100% graft survival at 7 months posttransplant [9]. Congruent with these reports, our study found similar patient and graft survival between 143 pediatric kidney recipients of COVID + donors and propensity score-weighted COVID − recipients, accounting for common clinical confounders. Our findings are consistent with adult UNOS studies reporting no difference in post-transplant patient and graft survival between COVID + and COVID − recipients [5, 6, 22, 23]. However, unlike our study, most adult studies reported 30-day or 1-year transplant outcomes.

We observed no difference in DGF rates and the length of hospital stay between COVID + and propensity score-weighted COVID − recipients. This mirrors the findings of an adult UNOS study of recipients transplanted between March 2020 and March 2023, which found no difference in the length of hospital stay or DGF rates between COVID + and COVID − recipients [7]. The absence of donor-derived COVID-19 transmission from non-pulmonary solid organ transplants provides a plausible explanation for the favorable findings associated with COVID + kidney transplants [22, 24, 25].

When we compared accepting a COVID + kidney to remaining on the waiting list for a COVID − offer, HR for mortality did not reach statistical significance. These results contrast with other pediatric studies, which indicate a survival benefit of accepting suboptimal kidneys, such as high KDPI kidneys or those donated after circulatory arrest [11, 12]. The lack of a survival benefit of a COVID + transplant versus remaining on the waiting list was likely due to the short follow-up period and the low number of events, underscoring the need for future studies with longer follow-up durations.

Our study had several limitations. Although our study provided the longest documented follow-up of pediatric COVID + recipients, the follow-up time was relatively short. Due to limited data granularity in the registry, we were unable to examine the effects of the timing and severity of donor COVID-19 infection, the presence of viral RNA in kidney tissue, or the type of SARS-CoV-2 variant on outcomes. Similarly, we could not evaluate the role of COVID vaccination in the safety of COVID + pediatric kidney transplants. Furthermore, data on transplant-mediated COVID-19 transmission and posttransplant antiviral treatment, such as remdesivir, was not available. Additionally, while we assessed acceptance rates, our study did not directly address center-level decision-making processes, which could provide valuable insights for future policy development. The retrospective nature of the study prevented adjustment for unknown confounders; however, the use of covariate-balancing propensity score weighting allowed for rigorous adjustment across a wide range of clinical factors. Despite limitations, this study provides important safety data for COVID + pediatric kidney transplants. Moreover, the population-based nature of the study ensures generalizability of the findings to the diverse population of the United States.

In conclusion, using the national SRTR registry, this study represents the most comprehensive analysis to date of COVID + deceased donor utilization in pediatric kidney transplantation. Our findings demonstrate a steady increase in the adoption of these donors across the United States, with initial outcomes showing no significant differences in DGF, hospital stay, or short-term graft and patient survival when compared to COVID − donors. These results provide pivotal baseline evidence supporting the viability of COVID + donors as a strategy to expand the donor pool for pediatric candidates. While these early indicators are highly encouraging, the novelty of this practice underscores the importance of ongoing surveillance. As this cohort matures, continued multi-center collaboration and extended longitudinal follow-up will be instrumental in building upon these findings. Such efforts will further refine our understanding of long-term results, ensuring the continued safety and optimization of transplant outcomes for children on the deceased-donor waiting list.

## Supplementary Information

Below is the link to the electronic supplementary material.

ESM 1 Graphical abstract (PPTX 159 KB)

## Data Availability

The data that support the findings of this study are available upon request to the corresponding author. These data were derived from the following resources available in the public domain: Scientific Registry of Transplant Recipients (https://www.srtr.org/).

## Abbreviations

COVID-19: Corona virus disease 2019
SARS-CoV-2 virus: Severe acute respiratory syndrome coronavirus 2
COVID +: Coronavirus disease positive
COVID −: Coronavirus disease negative
cPRA: Calculated panel reactive antibody
DGF: Delayed graft function
HLA: Human leucocyte antigen
HRSA: Health resources and services administration
KDPI: Kidney Donor Profile Index
OPTN: Organ Procurement and Transplantation Network
SRTR: Scientific Registry of Transplant Recipients
UNOS: United Network for Organ Sharing

## Declarations

### Disclaimer

The data reported here have been supplied by the United Network for Organ Sharing (UNOS) as the contractor for the OPTN. The interpretation and reporting of these data are the responsibility of the authors and in no way should be seen as an official policy of or interpretation by the OPTN or the U.S. Government. The content is solely the responsibility of the authors and does not necessarily represent the official views of the National Institutes of Health’s National Center for Advancing Translational Sciences.

## References

[1] Rees L (2009) Long-term outcome after renal transplantation in childhood. Pediatr Nephrol 24:475–484. 10.1007/s00467-007-0559-2 PMC2755795 17687572

[2] Lentine KL, Smith JM, Lyden GR, Miller JM , Booker SE, Dolan TG, Temple KR, Weiss S, Handarova D, Israni AK, Snyder JJ (2025) OPTN/SRTR 2023 annual data report: kidney. Am J Transplant 25:S22–S137. 10.1016/j.ajt.2025.01.020 PMC12414513 39947805

[3] Nimmo A, Gardiner D, Ushiro-Lumb I, Ravanan R, Forsythe JLR (2022) The global impact of COVID-19 on solid organ transplantation: two years into a pandemic. Transplantation 106:1312–1329 10.1097/TP.0000000000004151 PMC9213067 35404911

[4] OPTN evidence summary (2024) Donor SARS-CoV-2 testing & organ recovery considerations; low transmission risk to non-lung recipients when donors are SARS-CoV-2 NAT-positive under defined scenarios. https://www.hrsa.gov/sites/default/files/hrsa/optn/sars-cov-2-summary-of-evidence.pdf

[5] Yang J, Endo Y, Sasaki K, Schenk A, Pawlik TM (2024) Chronological and geographical variations in the incidence and acceptance of COVID-19–positive donors and outcomes among abdominal transplant patients. Clin Transplant 38:e15391. 10.1111/ctr.15391 38967586

[6] Bock MJ, Vaughn GR, Chau P, Berumen JA, Nigro JJ, Ingulli EG (2022) Organ transplantation using COVID-19-positive deceased donors. Am J Transplant 22:2203-2216. 10.1111/ajt.17145 PMC9349433 35822320

[7] Ji M, Vinson AJ, Chang S, Merzkani M, Lentine KL, Caliskan Y, Progar K, Nesselhauf N, Dubrawka C, Alhamad T (2023) Patterns in use and transplant outcomes among adult recipients of kidneys from deceased donors with COVID-19. JAMA Netw Open 6:e2315908. 10.1001/jamanetworkopen.2023.15908 PMC10230314 37252739

[8] Pizzo H, Soni PR, Nadipuram S, Garrison J, Jordan SC, Puliyanda D (2023) Utilization of SARS‐CoV‐2‐positive donors in pediatric renal transplantation. Pediatr Transplant 27:e14451. 10.1111/petr.14451 PMC9878121 36518031

[9] Clark JD, Albers EL, Albert JE, Berkman ER, Englund JA, Farris RWD, Johnson BA, Lewis‐Newby M, McGuire J, Rogers M, Thompson HM, Wagner TA, Wells C , Yanay O, Zerr DM, Limaye AP (2023) SARS‐CoV‐2 RNA positive pediatric organ donors: a case report. Pediatr Transplant 27:e14452. 10.1111/petr.14452 PMC9878170 36518025

[10] Feldman AG, Beaty B, Everitt M, Goebel J, Kempe A, Pratscher L, Danziger-Isakov LA (2023) Survey of pediatric transplant center practices regarding COVID-19 vaccine mandates for transplant candidates and living donors and use of COVID-19-positive deceased organs. Pediatr Transplant 27:e14513. 10.1111/petr.14513 PMC10509306 36939212

[11] Kizilbash SJ, Evans MD, Chinnakotla S, Chavers BM (2021) Use of expanded‐criteria donors and > 85 KDPI kidneys for pediatric kidney transplantation in the United States. Am J Transplant 21:1160–1170. 10.1111/ajt.16162 PMC7767891 32594613

[12] Kizilbash SJ, Evans MD, Chavers BM (2022) Survival benefit of donation after circulatory death kidney transplantation in children compared with remaining on the waiting list for a kidney donated after brain death. Transplantation 106:575-583. 10.1097/TP.0000000000003733 PMC8408288 33654002

[13] 13. Leppke S, Leighton T, Zaun D, Chen S, Skeans M, Israni AK, Snyder JJ, Kasiske BL (2013) Scientific registry of transplant recipients: collecting, analyzing, and reporting data on transplantation in the United States. Transplant Rev (Orlando) 27:50–56. 10.1016/j.trre.2013.01.002 23481320

[14] Austin PC (2011) An introduction to propensity score methods for reducing the effects of confounding in observational studies. Multivariate Behav Res 46:399–424. 10.1080/00273171.2011.568786 PMC3144483 21818162

[15] Imai K, Ratkovic M (2014) Covariate balancing propensity score. J R Stat Soc B Stat Methodol 76:243–263. 10.1111/rssb.12027

[16] Schaubel DE, Wolfe RA, Port FK (2006) Sequential stratification method for estimating the effect of a time-dependent experimental treatment in observational studies. Biometrics 62:910–917. 10.1111/j.1541-0420.2006.00527.x 16984335

[17] R Development Core Team (2012) R: a language and environment for statistical computing. R Foundation for Statistical Computing Vienna, Austria. https://www.R-project.org/

[18] Greifer N (2024) WeightIt: weighting for covariate balance in observational studies. R package version 1.3.2. https://cran.r-project.org/package=WeightIt

[19] Lumley T (2024) Survey: Analysis of complex survey samples. R package version 4.4. https://cran.r-project.org/package=survey

[20] Greifer N (2024) Cobalt: covariate balance tables and plots. R package version 4.5.5. https://cran.r-project.org/package=cobalt

[21] Asher J (2020) Transplantr: audit and research functions for transplantation. R package version 0.2.0. https://cran.r-project.org/package=transplantr

[22] Dhand A, Okumura K, Nabors C, Nishida S (2023) Solid organ transplantation from COVID positive donors in the United States: analysis of United Network for Organ Sharing database. Transpl Infect Dis 25:e13925. 10.1111/tid.13925 PMC9538265 35942924

[23] Schold JD, Koval CE, Wee A, Eltemamy M, Poggio ED (2022) Utilization and outcomes of deceased donor SARS‐CoV‐2–positive organs for solid organ transplantation in the United States. Am J Transplant 22:2217–2227. 10.1111/ajt.17126 PMC9350307 35730252

[24] Covarrubias K, Brubaker AL, Mekeel K, Pretorius V, Shah M, Adler E, Ajmera V, Schnickel GT, Aslam S (2023) Single-center experience on nonlung solid organ transplantation from SARS-CoV-2–positive donors. Transplantation 107:e41–e42. 10.1097/TP.0000000000004400 PMC9746228 36240442

[25] Koval CE, Poggio ED, Lin Y, Kerr H, Eltemamy M, Wee A (2021) Early success transplanting kidneys from donors with new SARS‐CoV‐2 RNA positivity: a report of 10 cases. Am J Transplant 21:3743–3749. 10.1111/ajt.16765 PMC8441915 34254424

